# Enhancing patient informed consent in elective skin cancer surgeries: a comparative study of traditional and digital approaches in a German public hospital

**DOI:** 10.1186/s12913-024-11225-3

**Published:** 2024-08-02

**Authors:** Alexandra Schulz, Sabine Bohnet-Joschko

**Affiliations:** 1 Chair of Healthcare Management and Innovation, Faculty of Management, Economics and Society, Witten/Herdecke University, 58455 Witten, Germany; 2 Schaeftlarnstrasse 66, 81371 Munich, Germany

**Keywords:** Shared decision making, Informed consent conversation, Patient informed consent, Patient engagement, Digitalisation, Patient satisfaction

## Abstract

**Background:**

This study aims to investigate the integration of modern sources of patient information, such as videos, internet-based resources, and scientific abstracts, into the traditional patient informed consent process in outpatient elective surgeries. The goal is to optimize the informed consent experience, enhance patient satisfaction, and promote shared decision making (SDM) between patients and surgeons. By exploring different patient informed consent formats and their impact on patient satisfaction, this research seeks to improve healthcare practices and ultimately enhance patient outcomes. The findings of this study will contribute to the ongoing efforts to improve the informed consent process in public hospitals and advance patient-centred care.

**Methods:**

Data collection occurred at the day care clinic of a prominent German public hospital, forming an integral component of a prospective clinical investigation. The study exclusively focused on individuals who had undergone surgical intervention for skin cancer. For the purpose of meticulous data examination, the statistical software SPSS version 21 was harnessed. In the course of this study, a chi-square test was aptly employed. Its purpose was to scrutinize the nuances in patient experiences pertaining to informed consent across four distinct categories, viz., oral informed consent discussion (Oral ICD), written informed consent discussion (Written ICD), video-assisted informed consent discussion (video-assisted ICD), and digitally assisted informed consent discussion (digital-assisted ICD). The primary dataset of this inquiry was diligently gathered via a structured questionnaire administered to a targeted cohort of 160 patients. Within this sample, a balanced representation of genders was observed, encompassing 82 males and 78 females. Their collective age span ranged from 18 to 92 years, with an average age of 71 years. A randomized selection methodology was employed to include participants in this study during the period spanning from July 2017 to August 2018.

**Results:**

Significant differences were observed across the groups for all research questions, highlighting variations in patient responses. Video-assisted and digital-assisted IC were rated as superior in patient satisfaction with information compared to written and oral IC. Demographic profiles of the four study groups were found to be comparable.

**Conclusion:**

The findings of this study indicate that the incorporation of digital technologies in the informed consent process can enhance patient understanding during outpatient elective skin cancer surgeries. These results have important implications for increasing patient satisfaction and improving the SDM process within the hospital environment.

**Supplementary Information:**

The online version contains supplementary material available at 10.1186/s12913-024-11225-3.

## Introduction

Efforts to promote patient-centred care in clinical practice play a pivotal role in enhancing healthcare quality [1]. In today’s healthcare landscape, patients are increasingly seeking active involvement in their healthcare decisions. *By embracing patient empowerment and respecting their autonomy, healthcare providers not only enhance patient satisfaction and build stronger patient-provider relationships but also fulfil their moral obligations, thereby contributing to the integrity of medical practice.* Central to this approach are SDM and informed consent, both highlighting the significance of involving patients in their own care. Recent research by Shahu et al. explores the quality variations in informed consent procedures, raising concerns regarding their alignment with patient-centred care and SDM [2]. These findings shed light on the need to critically evaluate and improve informed consent practices to better support patient-centred care and foster effective SDM.

### The critical role of patient informed consent and shared decision making in healthcare

Shared decision making (SDM) has become a cornerstone of patient-centred care, with the National Health Service (NHS) recognizing it as a fundamental principle [3]. This collaborative approach involves healthcare providers and patients making decisions together, considering quantified risks and patient preferences. It acknowledges that patients have unique values influencing their perception of risks and benefits, which differ from medical practitioners [2]. Patients contribute their values and healthcare goals, while doctors offer medical knowledge, training, experience, and judgment. The main objective of SDM is to achieve better health outcomes by fostering a patient-centred approach that emphasizes communication, leading to an agreement on the most suitable treatment option aligning with the patient’s goals and preferences [4]. SDM represents an advanced patient-centred care approach, involving collaborative decision-making between clinicians and patients [5]. It encourages consideration of multiple options, thoughtful deliberation, and identification of the best course of action based on the patient’s values and preferences [5, 6]. SDM promotes information sharing and mutual understanding, enabling patients and clinicians to explore different possibilities [2, 7]. By considering various options and tailoring care to individual needs, SDM strives to resolve the patient’s situation effectively [5].

#### Informed consent

, a legal requirement, involves clinicians disclosing treatment or procedure risks, benefits, and alternatives, allowing patients to accept or reject the proposed option [8]. The informed consent conversation is a critical element of SDM, especially in surgical procedures with multiple treatment alternatives and no definitive best choice [9]. Patients must fully understand the advantages and disadvantages of available treatment options to identify the most suitable course of action for their medical care. However, patient values, opinions, judgments, and outcome expectations may not always align with the surgeon’s expertise and strategy regarding potential outcomes [10, 11].

Patients must comprehend the risks and benefits associated with their decisions, actively participate in decision making by asking questions and expressing concerns, and feel empowered throughout the process. Physicians should ensure that patients receive all necessary information to make informed decisions and understand potential outcomes, ultimately enhancing patient engagement in the decision-making process [11]. Over time, the legal characterization of informed consent has preceded its ethical examination, with its qualifications expanding as the focus of medical decision making shifted from doctors to patients through a century of American court decisions [4, 12]. Informed consent establishes a legal foundation, obliging clinicians to communicate potential risks, benefits, and alternative options, allowing patients to make informed decisions about the most appropriate course of action [13]. However, current informed consent procedures often prioritize meeting legal standards and obtaining patient consent while overlooking the recognition of multiple sensible options for addressing the patient’s unique situation [12, 13]. This limitation reflects the technical and formal nature of informed consent, emphasizing administrative and legal requirements [14].

Patient informed consent is crucial in SDM, fostering trust, patient autonomy, transparency, and SDM within the healthcare system [15]. While informed consent is a legal requirement that varies across jurisdictions, its purpose extends beyond mere compliance. It entails providing patients with the necessary knowledge to ensure their understanding of the risks, benefits, and alternatives associated with their healthcare choices. Specific rules govern the process, including the provision of additional choices, the manner of presentation, and the documentation of consent in writing [14].

### Addressing gaps and challenges in patient informed consent and shared decision-making practices

In daily healthcare practice, informed consent often becomes a mere formality aimed at securing a patient’s signature, neglecting to explore all available options. Resource constraints limit patient involvement to passively accepting or rejecting a predetermined path, focusing more on legal requirements than on recognizing multiple sensible options for each patient [11, 12, 15]. This technical approach can overlook the need for comprehensive patient information, leading to reduced satisfaction and conflicts with the principles of shared decision making (SDM) [11]. Patients frequently express dissatisfaction with the way information is presented, its content, and the timing of discussions, highlighting the need for better communication about their condition, diagnosis, treatment options, and outcomes [15].

Despite supporting SDM, clinicians often fail to communicate the existence of multiple treatment options, limiting genuine patient involvement. To address these challenges, strategies that promote a comprehensive and patient-centred approach to informed consent are crucial. Incorporating modern information sources, such as videos, internet-based resources, and scientific abstracts, can help physicians convey complex concepts more effectively [11]. The limitations of informed consent procedures extend beyond the forms themselves and are embedded in the broader healthcare culture. Therefore, it is essential to ensure patients are well-informed, empowered, and actively involved in decision-making, enhancing patient engagement and care quality.

Bridging the gap between patient informed consent and SDM involves moving beyond a transactional approach to one that fosters collaboration. SDM should not be about merely obtaining consent for a predetermined action or placing the decision-making burden solely on the patient. Instead, it requires a collaborative effort that respects the patient’s values and preferences. This shift from a compliance-oriented focus to one that embraces open dialogue, active engagement, and mutual respect between healthcare providers and patients can enhance patient-centred care [16].

### Using modern technology to improve patient informed consent and shared decision-making

Traditionally, oral informed consent allows direct dialogue between healthcare providers and patients but lacks a persistent reference for patient comprehension in complex medical scenarios. Written informed consent enhances patient recall and understanding by providing a tangible document for future reference, combining detailed documentation with personal interaction [17, 18]. This hybrid approach of written materials and oral explanations yields the best outcomes by catering to diverse patient needs and learning styles.

Recent advancements in digital technology have significantly improved the informed consent process, enhancing patient understanding and satisfaction. Digital tools increase patient engagement and comprehension, transitioning from paper-based to interactive, patient-centred methods [19, 20]. Studies show that multimedia digital tools outperform traditional methods in improving patient comprehension and satisfaction by presenting information in an accessible format [19]. These tools are particularly effective in surgical settings, offering detailed and customized patient education that fosters informed decision-making and patient autonomy [21]. The development of digital informed consent apps highlights the potential and challenges of new formats in public health research. These apps streamline the consent process with tailored educational content and interactive features, engaging patients more effectively. However, ensuring data security, maintaining patient privacy, and addressing the digital divide are crucial challenges that must be managed [22].

The broader integration of digital technologies in informed consent shifts patient engagement from passive recipients to active participants in treatment planning and decision-making [23]. E-health innovations advance patient autonomy and improve the quality of informed consent, helping patients make well-informed decisions that reflect their health goals and preferences, ultimately enhancing patient outcomes [24].

### Study objectives: bridging the gap in patient informed consent and shared decision making through innovative approaches

The study explores enhancing traditional informed consent through digital tools in elective skin cancer surgeries, filling a literature gap by systematically comparing digital and traditional methods’ impact on patient satisfaction and understanding. Unlike broader studies, this research focuses on patients undergoing a specific surgical procedure, providing detailed, applicable conclusions for similar clinical environments. The study compares various informed consent formats—oral, written, video-assisted, and digitally assisted—to identify which most effectively enhance patient comprehension and satisfaction. Clear informed consent is crucial in complex cancer treatments where decisions significantly affect patient quality of life.

## Methods

### Research question

The primary objective of this study was to evaluate the effect of various informed consent formats on patient satisfaction in outpatient surgical settings. The research specifically aimed to investigate whether augmenting traditional informed consent methods with additional, format-specific information could enhance patient satisfaction and deepen their understanding of the surgical interventions they were to undergo. This investigation was guided by the hypothesis that different modalities of informed consent—oral, written, video-assisted, and digitally assisted—differ significantly in their capacity to improve patient comprehension and participation. Key indicators used to measure patient satisfaction included the clarity of information regarding medical conditions and treatment options, patients’ perceived involvement in decision-making processes, and their comfort with the information provided. The secondary objective was to ascertain which of these informational mediums most effectively aligns with patient preferences, thus offering a more customized and effective approach to informed consent in outpatient surgical contexts.

### Study material

In collaboration with Thieme Compliance GmbH, a patient education video titled “Skin cancer: Removal and Subsequent Wound Care” was created. Based on this video, a paper-based informed consent form and a digital web-based online information page were developed. Both media included all relevant images and the complete verbal and written communication presented in the video. Thus, in addition to the oral informed consent discussion, three additional media were created: written informed consent (Written IC), video-assisted informed consent (video-assisted IC), and digitally assisted informed consent (digital-assisted IC).

### Methodology of study questionnaire design

In our study, we focused on the development of a questionnaire aimed at measuring satisfaction in the context of our outpatient skin cancer surgery. To achieve this, we compiled a questionnaire consisting of 10 questions that were sourced from existing questionnaires previously validated and deemed reliable. These questions were carefully selected from articles published in peer-reviewed journals, ensuring the integrity and scientific foundation of our instrument [25–29].

The process of conceptualization in the questionnaire development involves meticulously designing, developing, and adapting questions to precisely capture data aligned with specific research objectives. This process typically includes the incorporation and modification of questions from previously validated questionnaires, significantly enhancing the reliability and validity of the new questionnaire [30–32]. A thorough literature review was conducted to identify existing questionnaires that had demonstrated validity, cited in peer-reviewed publications. Validated questions that were relevant to our research questions were selected. These questions underwent a detailed validity assessment to ensure their appropriateness and alignment with the leading research questions of the current study. Adaptations were then made both linguistically and contextually to tailor the questions to the specific objectives of our study and the characteristics of the study group.

To assess the comprehensibility and reliability of the adapted questions, a pilot study was conducted involving 10 participants. This preliminary survey allowed us to gather initial feedback and make necessary adjustments to the questionnaire.

We developed a questionnaire consisting of 10 questions to assess patient satisfaction in the context of our outpatient skin cancer surgery. (see Table 1).

### The patient information regarding my upcoming surgery (including all materials used and the medical briefing …

**Table 1:** Study questionnaire, 7 likert – scale: 1 = applies to a small extent; 7 = applies completely

Q1	Helped me understand the condition.	1	2	3	4	5	6	7
Q2	Helped me understand the treatment options.	1	2	3	4	5	6	7
Q3	Reduced my concerns about my condition.	1	2	3	4	5	6	7
Q4	Gave me courage.	1	2	3	4	5	6	7
Q5	Gave me hope that I can feel better again.	1	2	3	4	5	6	7
Q6	Helps me participate in treatment decisions.	1	2	3	4	5	6	7
Q7	Showed me how I can contribute to the success of the treatment myself.	1	2	3	4	5	6	7
Q8	Encouraged me to be proactive in improving my condition.	1	2	3	4	5	6	7
Q9	Explained the treatment steps in detail.	1	2	3	4	5	6	7
Q10	Explained the post-discharge treatment thoroughly.	1	2	3	4	5	6	7

### Study design

This study examined patients who were undergoing surgical treatment for skin cancer within the ambulatory setting of a German public hospital. It was structured as a prospective clinical trial. Prior to their procedures, all participants underwent an informed consent discussion led by the treating physician.

Participants were randomly allocated into one of four groups to assess the impact of different additional informational aids on their understanding and engagement. The control group received solely the standard oral informed consent discussion. The second group received written patient information before the oral discussion to facilitate preparation for the informed consent process (Written IC). The third and fourth groups were provided with supplemental information through a patient information video (Video-assisted IC) and access to a patient information webpage (Digital-assisted IC), respectively, also prior to the oral discussion.

After reviewing the informed consent materials, each patient participated in a structured consultation with their physician. This process ensured an interactive and informed dialogue, which is crucial for effective SDM. This study design aimed to evaluate how different formats of pre-discussion information could enhance patient understanding and participation in their care decisions.

### Data analysis method

In this study, we aimed to investigate the variations in patient informed consent experiences among four groups (Oral ICD, Written ICD, Video-Assisted ICD, and Digital-Assisted ICD) and their impact on patient satisfaction. For data analysis, we utilized SPSS version 21, employing various statistical tests including the Chi-Square test, Kruskal Wallis test, and Man Whitney U test.

### Ethical considerations

This prospective clinical study was conducted at a single center and received ethical approval from the Ethical Review Committee of the University of Witten Herdecke, Germany (protocol number 13/2017). Prior to participation, all patients provided complete written informed consent. The study included patients who underwent excision of a basal cell or squamous cell carcinoma in the outpatient clinic of the department of plastic surgery between July 2017 and August 2018. Individuals who were mentally incapacitated, prisoners, and incarcerated patients were excluded from the study. The research methodology adhered to the principles outlined in the Helsinki Declaration.

## Results

### There is no significant demographic difference among the four study groups

In Table 2, we present the demographics of the study group. The Chi-Square test was conducted to examine differences between the groups (Oral ICD, Written ICD, Video-assisted ICD, and Digital-assisted ICD) for each of the seven variables measured. The results indicated no significant differences among the groups.

Demographically, the groups were similar in terms of gender distribution, with varying mean ages ranging from 67.65 to 72.05 years.

Regarding treatment history, the Oral ICD group had a higher proportion of new clinic attendees (23 out of 40) compared to the other groups. The Written ICD group had the highest number of participants with prior treatments (14 out of 40).

In terms of healthcare utilization, the Oral ICD group had the highest average number of hospitalizations in the last 5 years (1.85), followed closely by the Digital-assisted ICD group (1.50). Clinic operations and department-specific operations showed similar patterns across the groups, with the Oral ICD group having slightly higher averages.

Notably, complications were reported by one participant in both the Oral ICD and Digital-assisted ICD groups, while the other groups had no reported complications.

Overall, while there were some variations in treatment history and healthcare utilization, the groups exhibited comparable demographic characteristics, suggesting a balanced comparison in the study. The lack of significant differences in the Chi-Square test further supports the comparability of the groups across the variables examined.

**Table 2:** Demographics of the study group

Chi-square test for differences between the groups.	Variables	Oral IC	+ written IC	+ video-assisted IC	+ digital-assisted IC
0.729	Gender	18 m, 22 f	20 m, 20 f	21 m, 19 f	17 w, 23 f
0.797	Age	mean 70,68 (min 18, max 91, Std. 15,252)	mean 72,05 (min 47, max 88, Std. 9,987)	mean 71,88 (min 30,max 92, Std. 13,674)	mean 67,65 (min 25, max 88, Std.16,964)
0.606	Have you already been treated by us?	23 no, 17 yes	26 no, 14 yes	20 no, 20 yes	23 no, 17 yes
0.121	Number of surgeries performed in our clinic so far.	mean 1,85 (min 0, max 9, Std. 2,2,48)	mean 1,13 (min 0, max 5, Std. 1,159)	mean 1,50 (min 0, max 8, Std.1,710)	mean 1,50 min 0, max 11, Std.2,375)
0.261	Number of surgeries performed in our clinic to date.	mean 1,55 (min 0, max 9, Std. 2,012)	mean 1,10 (min 0, max 10, Std.1,823)	mean 1,02 (min 0, max 10, Std. 1,776)	mean 1,08 (min 0, max 5, Std.1,439)
0.282	Number of surgeries for the same condition in our department so far.	mean 1,50 (min 0, max 9, Std.2,088)	mean 0,95 (min 0, max 10, Std. 1,753)	Mean 0,90 (min 0, max 10, Std. 1,736)	mean 1,23 (min 0, max 9, Std.1,888)
0.567	Complications	39 no, 1 yes	40 no	40 no	39 no, 1 yes

### There are significant differences across all four study groups in response to the 10 research questions

In Table 3, we present the results of the Kruskal-Wallis test assessing the statistical differences among the four study groups. We conducted this test to compare the responses of the four study groups across all 10 study questions for significant differences. The outcomes indicated significant differences among the groups for all assessed questions.

**Table 3:** Kruskal-Wallis test assessing the statistical differences among the four study groups

	Q1	Q2	Q3	Q4	Q5	Q6	Q7	Q8	Q9	Q10
Chi-Square	42.606	35.983	42.253	47.170	46.630	37.857	44.400	51.061	38.941	37.923
Degrees of freedom	3	3	3	3	3	3	3	3	3	3
Significance level	0.000	0.000	0.000	0.000	0.000	0.000	0.000	0.000	0.000	0.000

### Incremental gains in patient satisfaction and understanding across diverse informed consent formats

Based on the hypothetical mean Likert scale scores for each informed consent group and question provided, several conclusions can be drawn about patient satisfaction and understanding in the study. The scores reveal a moderate level of satisfaction and understanding for oral informed consent (Oral IC) with values ranging from 3.0 to 3.5. This level improves with written informed consent (Written IC), which shows better outcomes with scores between 4.0 and 4.3. The satisfaction and understanding further increase in the video-assisted informed consent (Video-assisted IC) group, with scores from 4.5 to 4.8, suggesting that the visual and auditory components are effective. The highest satisfaction and understanding are observed in the digitally assisted informed consent (Digital-assisted IC) group, with scores peaking between 4.8 and 5.0, likely due to the interactive and user-friendly nature of digital tools. The data indicate a clear trend that as the medium of informed consent transitions from traditional oral methods to modern digital approaches, patient satisfaction and understanding significantly improve. This enhancement is particularly pronounced with digital and video formats, which excel in providing higher mean scores. These formats seem to be more effective at conveying complex medical information, enhancing the informed consent process. Moreover, the variability in responses across the groups highlights that digital-assisted and video-assisted formats are more effective in meeting patient needs for information about their healthcare decisions. In contrast, oral and written consents, while traditional, show a need for integration with more interactive tools or supplementary visual aids to boost understanding and satisfaction.

### Between which of the study groups do we find significant differences for which research question? Pairwise comparison of 10 research questions among each single study group

Next, a pairwise comparison was conducted employing the Man Whitney U test between each of the four study groups using the 10 research questions to uncover significant differences. The comparison yielded the following findings:

#### Oral versus written informed consent

Significant differences favouring written informed consent were observed in questions Q3 to Q8, and Q10, indicating better patient outcomes in areas such as reducing concerns, fostering courage, hope, and proactive involvement in treatment decisions (*p* < 0.05). No significant differences were noted in questions Q1, Q2, and Q9, suggesting similar effectiveness in helping patients understand their condition and the treatment options, and explaining treatment steps in detail (*p* > 0.05).

#### Oral versus video-assisted informed consent

Video-assisted informed consent demonstrated superior efficacy across all assessed questions (*p* < 0.001), suggesting that the visual and auditory components significantly enhance patient understanding and engagement.

#### Written versus video-assisted informed consent

Similar to oral versus video-assisted, video-assisted consent showed significantly better outcomes in all questions when compared to written consent (*p* < 0.001), reinforcing the effectiveness of multimedia elements in patient communication.

#### Written versus digital-assisted informed consent

Digital-assisted informed consent outperformed written consent in most areas, with significant differences observed in questions Q1, Q2, Q4 to Q8, Q9, and Q10 (*p* < 0.05). The interactive nature of digital tools likely contributed to better understanding and engagement, except for Q3 (“Reduced my concerns about my condition”), where no significant difference was found.

#### Video-assisted versus digital-assisted informed consent

Differences between these two advanced methods were less pronounced. Significant differences were found in questions Q1 and Q5 only, suggesting specific areas where digital tools may offer additional benefits in understanding the condition and fostering hope. No significant differences were noted in the other questions, indicating a generally high level of efficacy for both methods in enhancing patient engagement and understanding.

### Which informed consent media is statistically superior in terms of satisfaction with patient information?

The following graph demonstrates, which study group is superior compared to the other groups for each single study question. The findings are derived from the results of the pairwise comparison conducted using the Mann-Whitney U test, as well as the average value of the absolute scores obtained from the survey responses pertaining to the 10 research questions in each group.

### In the present study, no superiority of oral informed consent (IC) was observed over any other IC process examined

**Fig. 1:**
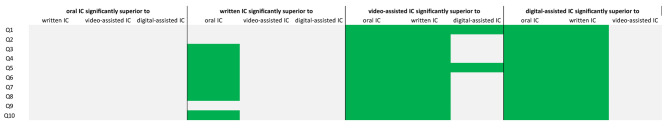
Video and digital-assisted informed consent media outperform written and oral methods in study findings

In Fig. 1, the graph presented illustrates the findings of the study, highlighting the relative superiority of different informed consent (IC) media in terms of satisfaction with information. Specifically, the results indicate that video-assisted and digital-assisted IC media were consistently rated as superior to written IC and oral IC alone. Notably, oral IC did not demonstrate superiority over any of the examined media in the study. On the other hand, written IC was found to be superior only to oral IC.

## Discussion

The objectives of the study, titled “Enhancing patient informed consent in elective skin cancer surgeries: a comparative study of traditional and digital approaches in a German public hospital,” are centred on investigating the integration of modern sources of patient information, such as videos, internet-based resources, and scientific abstracts, into the traditional patient informed consent process for outpatient elective surgeries. The study aims to optimize the informed consent experience, enhance patient satisfaction, and promote SDM between patients and surgeons. By exploring different patient informed consent formats and their impact on patient satisfaction, this research seeks to improve healthcare practices and ultimately enhance patient outcomes.

The findings of this research underscore the importance of diversifying patient informed consent materials beyond traditional written forms. By incorporating digital platforms, videos, and oral discussions, patients’ understanding and engagement can be significantly enhanced. Our findings demonstrate that video-assisted and digital-assisted patient information, alongside oral and written consent, significantly improve the overall informed consent process, leading to greater patient satisfaction and a more comprehensive understanding of the treatment options.

### Clarity and easy access

#### Informed consent

often faces two common challenges: illegible documents and rushed decisions. Illegible documents can impede comprehension, especially for patients with limited healthcare familiarity. To improve clarity, it is advisable to provide information in written form. Additionally, patients may feel pressured and have insufficient time for inquiries before undergoing a surgery. To address these issues, it is recommended that informed consent be presented with ample time for patients to consider the procedure and seek clarification by asking questions [2]. Patients should give their consent voluntarily and without any external pressure. Additionally, patients should have the freedom to accept or refuse the suggested treatment or procedure [2, 8]. Video and digital formats provide patients with user-friendly interfaces and offer accessibility and convenience. Patients can access the information at their preferred pace, review it multiple times as required, and even revisit it at a later time. This flexibility empowers patients to engage with the information when they are most receptive and caters to their individual learning preferences.

### [443–470] consistency and standardization

In the realm of patient information, it is essential to assess its quality based on both content and structure. Content-wise, patient information should encompass a comprehensive description of the medical condition, present all available treatment options along with their detailed consequences, provide a list of credible sources for the information presented, and allow space for patients to ask questions. The content should be well-balanced, grounded in evidence, up-to-date, visually illustrated, and easily comprehensible. Additionally, it should be tailored to suit the specific needs and characteristics of the target audience [26]. Videos and digital platforms offer valuable means of delivering consistent and standardized information to patients. By structuring the content and ensuring accuracy, these tools minimize variations in information provided during oral discussions, reducing misunderstandings and enhancing the effectiveness of informed consent. Additionally, they provide greater control over information presentation, enabling adherence to guidelines and facilitating the comparison of treatment options for improved decision-making by both patients and healthcare providers. Research has demonstrated that these formats improve patient recall and comprehension beyond what can be achieved through oral discussions alone. The visual and interactive elements of videos and digital platforms facilitate information processing and retention, resulting in a deeper understanding of the treatment options and potential outcomes [33].

In our study, we employed a series of questions extracted from validated questionnaires that have been previously published in peer-reviewed journals. These questions were specifically chosen for their ability to assess patient satisfaction and their understanding of treatment-related information. Notable questions included those that helped patients understand the condition and treatment options, as well as questions that detailed treatment steps and post-discharge care. The inclusion of these questions aimed to evaluate the effectiveness of the provided information in reducing misunderstandings and enhancing patients’ comprehension of their medical condition and the treatments involved. This methodology ensured a thorough assessment of how well information clarity impacted patient knowledge and satisfaction. Related studies have further explored the constructs and quality assessments of online health information, showing significant impacts on patient decision-making [34–36].

### Empowerment and SDM

Previous studies have indicated that providing comprehensive information about planned treatments can lead to satisfactory patient participation in treatment decision-making (Shahu, Schwartz et al., 2017; Bernat and McQuillen, 2021). Extensive research has consistently shown that SDM contributes to improved patient outcomes, such as higher treatment adherence, better understanding of health risks, and increased patient satisfaction [5, 6, 8].

Our study findings indicate that integrating video-assisted and digital-assisted informed consent with the traditional oral method improves patient satisfaction with the information provided. The inclusion of visual representations has been demonstrated to enhance comprehension and memory compared to oral discussions alone. Further research in the future will be necessary to examine the impact of these digital and video-based information mediums on SDM. The focus of the investigation should be to which extend the use of these media helps patients better understand their health situation, consider various treatment options, and personalize their care accordingly.

### Healthcare shifts to patient-centric decision-making for competitive advantage

In the evolving landscape of healthcare, hospitals and healthcare organizations are increasingly recognized not merely as centers of medical expertise, but also as service providers tasked with meeting the heightened expectations of an informed patient populace. Today’s healthcare consumers are more assertive and demanding, seeking greater involvement in their treatment choices and access to comprehensive information about their care options. This shift necessitates a transformation in how hospitals operate, emphasizing the importance of patient engagement and SDM [37]. To stay competitive, healthcare providers must adapt to these accelerating demands by fostering environments where patients are viewed as integral partners in the decision-making process [38]. This approach not only enhances patient satisfaction and outcomes but also positions these institutions at the forefront of a patient-centred healthcare revolution [39].

### Limitations

The study’s generalizability is limited due to its single-hospital focus, specific medical indication, and single-country setting. To improve generalizability, future research should replicate the study with a larger and more diverse patient population, including different healthcare facilities and a broader range of medical conditions. A multicentre international study design would help explore cultural and regional variations. Broadening the research scope will lead to a more comprehensive understanding, stronger external validity, and more robust conclusions. These efforts are essential for advancing health services research and guiding evidence-based clinical decision-making. We encourage the conduct of additional studies to investigate the quality of standardization of patient information in our study. Further research is needed to comprehensively assess and enhance the effectiveness of information dissemination within the context of patient informed consent processes. Furthermore, it is very interesting for further research to examine the direct influence of patient satisfaction on SDM within a comparable patient cohort.

## Conclusion

As part of the ongoing efforts to promote patient-centred care, the informed consent procedure plays a crucial role in facilitating safe, efficient, and high-quality decision-making in healthcare. It is imperative to establish standardized patient-centred protocols for informed consent and encourage healthcare systems to consistently adhere to these protocols for every procedure.

The integration of digital technologies into the informed consent process has the potential to enhance patient understanding and engagement, ultimately improving the delivery of healthcare services. These findings expand our knowledge of the advantages and implications of digital-assisted informed consent in healthcare settings, emphasizing the potential of technology to optimize the consent process.

### Electronic supplementary material

Below is the link to the electronic supplementary material.

Supplementary Material 1

## Data Availability

The datasets utilized and/or analysed in the present study are available from the corresponding author upon reasonable request.
