# Supplementary material for: Enhancing patient informed consent in elective skin cancer surgeries: a comparative study of traditional and digital approaches in a German public hospital
Source: BMC Health Serv Res. 2024 Aug 2;24:879. doi: 10.1186/s12913-024-11225-3 (PMC11295654; doi:10.1186/s12913-024-11225-3)
Supplement: Supplementary file 1 — Supplementary Material 1 [file 12913_2024_11225_MOESM1_ESM.pdf]

The patient information regarding my upcoming surgery (including all materials used and the medical briefing…

Q1	Helped me understand the condition.	1	2	3	4	5	6	7
Q2	Helped me understand the treatment options.	1	2	3	4	5	6	7
Q3	Reduced my concerns about my condition.	1	2	3	4	5	6	7
Q4	Gave me courage.	1	2	3	4	5	6	7
Q5	Gave me hope that I can feel better again.	1	2	3	4	5	6	7
Q6	Helps me participate in treatment decisions.	1	2	3	4	5	6	7
Q7	Showed me how I can contribute to the success of the treatment myself.	1	2	3	4	5	6	7
Q8	Encouraged me to be proactive in improving my condition.	1	2	3	4	5	6	7
Q9	Explained the treatment steps in detail.	1	2	3	4	5	6	7
Q10	Explained the post-discharge treatment thoroughly.	1	2	3	4	5	6	7

Study questionnaire, 7 Likert – Scale: 1= applies to a small extent; 7= applies completely
